# The effects of an educational intervention based on the protection motivation theory on the protective behaviors of emergency ward nurses against occupational hazards: a quasi-experimental study

**DOI:** 10.1186/s12912-024-02053-1

**Published:** 2024-06-18

**Authors:** Mohadeseh Nouri, Saeed Ghasemi, Sahar Dabaghi, Parvin Sarbakhsh

**Affiliations:** 1 Student Research Committee, Department of Community Health Nursing, School of Nursing and Midwifery, Shahid Beheshti University of Medical Sciences, Tehran, Iran; 2 Department of Community Health Nursing, School of Nursing and Midwifery, Shahid Beheshti University of Medical Sciences, Tehran, Iran; 3 Department of Statistics and Epidemiology, Faculty of Health, Tabriz University of Medical Sciences, Tabriz, Iran

**Keywords:** Education, Emergency wards, Health behaviors, Nurse, Occupational health, Quasi-experimental study

## Abstract

**Background:**

Emergency ward nurses face a variety of occupational hazards due to the nature of their occupational and professional duties, which can negatively affect their health. Therefore, this study aimed to evaluate the effects of an educational intervention based on the protection motivation theory on the protective behaviors of emergency ward nurses against occupational hazards in Tehran, Iran, in 2023.

**Methods:**

The present quasi-experimental study was conducted with two intervention and control groups, using a pretest-posttest design. A total of 124 nurses working in the emergency wards of four hospitals (two hospitals for the intervention group and two hospitals for the control group by random assignment) were selected by multistage sampling method. The educational intervention based on the protection motivation theory was implemented for the intervention group for three weeks. The nurses of both groups completed a demographic questionnaire and the scale of emergency ward nurses’ protective behaviors against occupational hazards before, immediately, and one month after the intervention. Data analysis was performed using descriptive and inferential methods.

**Results:**

The two groups were similar in terms of demographic characteristics at the baseline (*p* > 0.05). Protective behaviors of emergency nurses against occupational hazards and their sub-scales (physical, chemical, biological, ergonomics, and psychosocial hazards) were higher in the intervention group than in the control group immediately and one month after the educational intervention. In addition, the measurement over time also showed the positive effect of time and educational intervention on the protective behaviors of emergency nurses against occupational hazards and their sub-scales in the intervention group.

**Conclusion:**

These findings showed that the educational intervention based on the protection motivation theory can be effective and helpful in improving the protective behaviors of emergency ward nurses against occupational hazards and their sub-scales. Future studies can focus on a more specific design of this kind of intervention based on the type of occupational hazards and needs of nurses in different wards.

**Supplementary Information:**

The online version contains supplementary material available at 10.1186/s12912-024-02053-1.

## Background

The most occupational hazards for HealthCare Workers (HCWs), including emergency ward nurses are physical, chemical, biological, ergonomic, and psychosocial hazards [1–3]. Emergency ward nurses face various occupational hazards while performing their duties, and the safety of nurses and patients depends on the nurses’ knowledge of these hazards and appropriate protective behavior [4].

Physical hazards include exposure to extreme temperatures, tripping, slipping, cuts, falling, various radiations, unusual noise, electric shock, fire, and explosions [1–3]. The results of one study in Egypt showed that most nurses (62.4%) had poor knowledge about physical occupational hazards [5].

Chemical hazards, including exposure to cleaning and disinfecting agents, sterilant materials, mercury, toxic drugs, pesticides, latex and laboratory chemicals and reagents; this hazard may lead to poisoning, allergic reactions, dermatitis, cancer, and maternal health effects, which may occur during compounding, unpacking, cleaning the environment, etc [1–3]. A systematic review study had shown that the incidence of occupational contact dermatitis for some groups of HCWs was high [6].

Biological hazards include exposure to blood-borne and air-borne pathogens; such as Hepatitis B virus (HBV), Hepatitis C virus (HCV) and Human immunodeficiency virus (HIV), tuberculosis, etc [1–4]. The results of another systematic review also showed a high prevalence of needle stick injuries among HCWs, and health services related to this regard should be improved [7].

Ergonomic hazards include the inappropriate design of the work environment, inappropriate position while working, and repetitive procedures, which may lead to musculoskeletal disorders [1–3]. In a study in Malaysia, almost all nurses (97.3%) had work-related musculoskeletal disorders during the past year, so this problem should be considered seriously [8]. In another study in Saudi Arabia, 85% of nurses participating in the study reported at least one musculoskeletal disorder, which was associated with factors such as hours of working and the weight of nurses [9].

Psychosocial hazards include stressful conditions, work environment violence, job strain, burnout, exhausting work shifts, long working hours, loss of reputation, being threatened and bullied by colleagues, interpersonal communication at the work environment, satisfaction with the job and imbalanced roles and responsibilities [1–3]. In a study, some problems and stressors faced by nurses working in the emergency ward were burnout, workplace violence, moral distress, chaotic work environment, etc [10]. The results of the study in the United States of America (USA) showed that the psychosocial job stress of emergency ward nurses was prevalent [11]. Another study in Kenya on emergency nurses also revealed a high prevalence of violence in the workplace; 81.7% and 73.2% for lifetime and one year respectively, and this is a significant problem [12].

Social and behavioral theories can be useful for designing educational interventions to improve the protective behaviors of HCWs against occupational hazards [13]. Protection motivation theory (PMT) is one of these theories, was introduced by Rogers in 1975, and since has been widely adopted as a framework for the intervention in health-related behavior [14]. The results of a study indicated that education based on the constructs of the PMT increased the protective behaviors of medical laboratories’ staff [15]. The results of another study, indicated that educational intervention based on the PMT increased the preventative behaviors of a group of hospital staff against respiratory infections [16]. Some other types of studies have been done on people with jobs and professions other than the health systems and HCWs; for example, the results of a study indicated the effectiveness of an educational intervention based on the PMT in promoting the protective behaviors of farmer’s ranchers against brucellosis [17] and the employees of governmental offices against COVID-19 [18]. These types of interventions sometimes were not effective in changing the protective and healthy behaviors of other people in other contexts [19, 20]. Considering the above-mentioned literature; occupational hazards and protective behaviors of emergency nurses against them are important issues in health systems, and PMT is a tool for designing and implementing educational interventions that may promote the protective behaviors of these HCWs against occupational hazards and sufficient scientific evidences were not found in this field by research team; so this study aimed to evaluate the effects of an educational intervention based on the PMT on the protective behaviors of emergency ward nurses against occupational hazards.

## Methods

### Research design and setting

This quasi-experimental study was conducted on two groups, intervention and control, using the pretest-posttest design among emergency ward nurses of four educational hospitals (two hospitals for each group by random allocation) in Tehran, Iran, in 2023.

### Sample size and sampling methods

**Fig. 1:**
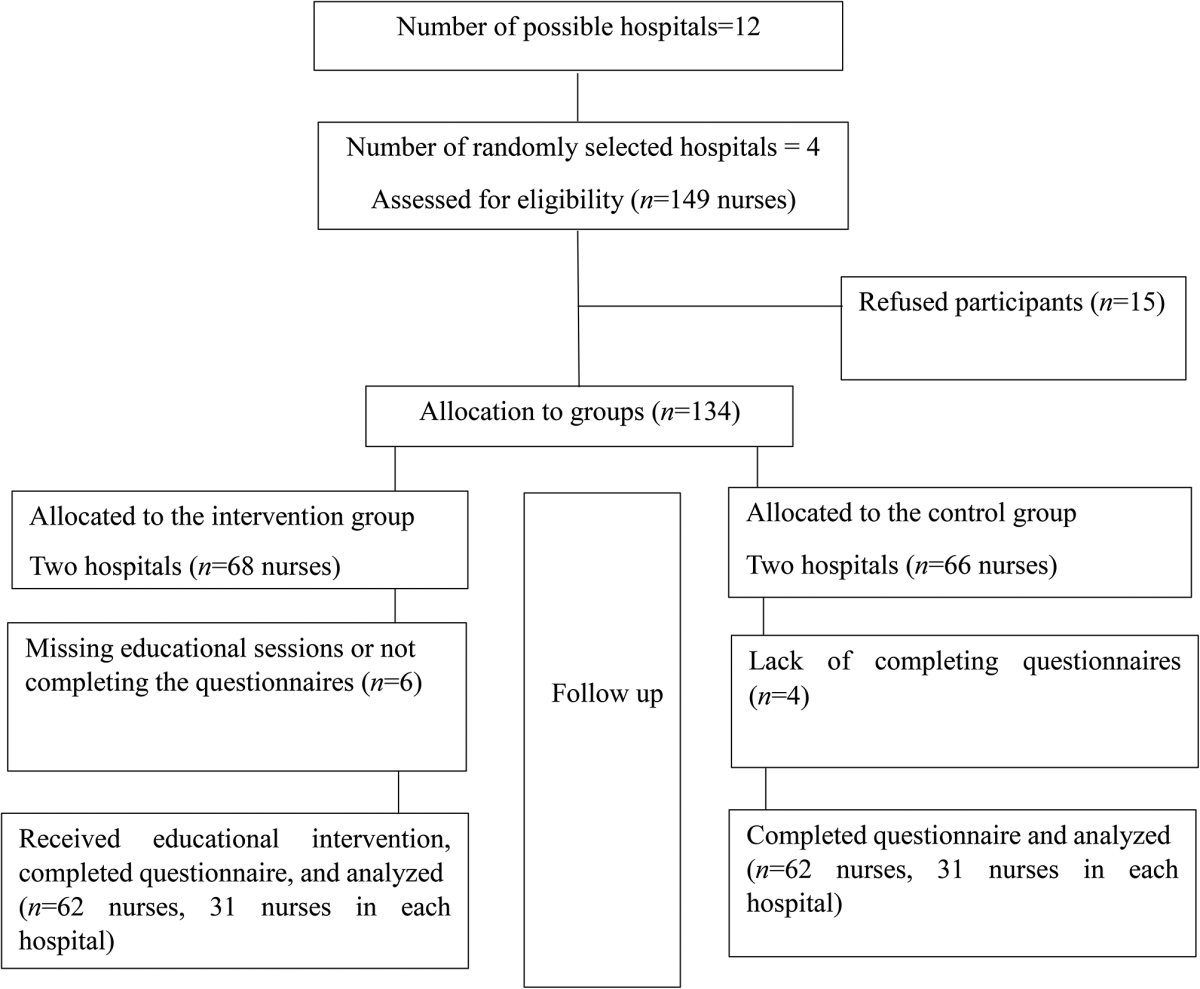
Consort diagram

The sampling method of this study was multistage. To prevent the transfer of information between the intervention and the control groups, randomization was performed at the hospital level. So, from 12 possible educational hospitals, because of the executive ability and study facilities, four hospitals were randomly selected by lottery and of them, two hospitals were randomly assigned for each intervention and control group. After estimating the number of the required nurses for each hospital, nurses who had inclusion criteria were selected using convenience sampling. The emergency nurses who had exclusion criteria were excluded from the study and replaced by other nurses from the same hospital. This process continued until data collection was completed. Ultimately, 31 nurses from each hospital, 62 nurses in each group, and a total of 124 nurses were enrolled (Fig. 1).

The total sample size for this cluster randomized study was based on 90% power, 95% confidence, estimation of the standard deviation and the effect size greater than or equal to 20% improvement in self-efficacy due to the educational intervention according to the similar study [21], considering two hospitals for intervention group and two hospitals for control group, and considering ICC = 0.2, the total sample size was calculated to be *n* = 62 emergency nurses for each group (31 nurses had to be recruited from each hospital).

Inclusion criteria were as follows: verbal and written informed consent, desire to participate in the study, and having appropriate communication status to participate in the study. Exclusion criteria were failure to complete the questionnaires, missing more than one of the education sessions in the intervention group, translocation to other wards during the study, and participation in similar training courses.

### Intervention group procedure

The educational content of the intervention used in this study covered almost all topics about occupational hazards for emergency ward nurses, prepared and extracted from the relevant literature [1–4, 14, 22] and the experiences of the research team. The initial educational content was evaluated by three experts outside the research team. These evaluators had ph.D. degrees in nursing and were faculty members of the Department of community health nursing of Shahid Beheshti University of Medical Sciences, whose opinions were re-evaluated and applied by the research team if needed. Finally, the educational content was confirmed by the three experts and the research team.

The educational intervention in this study was prepared based on constructs of the PMT (Protective behaviors, intention, perceived severity, perceived vulnerability, fear, response costs, rewards of maladaptive response, self-efficacy, and response efficacy) (Table 1).

**Table 1:** Educational sessions and content offered to the participants of the intervention group

Educational sessions (Time)	Educational content	Constructs of PMT	Instructors	Training location	Training methods and materials
1(60 min)	Acquaintance with various occupational hazards and diseases, and theirs’s possible complications for the nurse and her/his family	**Perceived vulnerability**: The emergency ward nurses were educated and reminded that all nurses in this ward were vulnerable to physical, chemical, biological, ergonomic, and psychosocial hazards. **Perceived Severity**: Education about the possibility of adverse effects (for example, acute back pain related to ergonomic hazards) or very adverse effects (fatal diseases such as HIV, HBV, and HCV) of occupational hazards for nurses and their families. **Fear**: Statistics about occupational hazards and diseases among nurses and HCWs were presented. The fears and concerns of nurses in the field of occupational hazards and diseases were discussed.	First author	Quiet places in the emergency ward	Multimethod; lecture (face-to-face presentation with electronic slide PowerPoint, PDF files, question and answer), indirect training including transfer of these files and pamphlets to nurses’ mobile phones via Email, Bluetooth, WhatsApp, Telegram App, etc.
2 (45 min)	Physical, chemical, and biological hazards were discussed in more detail, and protective strategies against these hazards were educated.	**Protective behaviors**: Protective behaviors against physical, chemical, and biological hazards were taught to emergency ward nurses. **Intention**: The objective was to increase the motivation of nurses to comply with safety instructions and protective behaviors against physical, chemical, and biological hazards. **Self-efficacy**: Nurses were educated on how to perform protective behavior. For example, what should be done after a needle stick? **Response efficacy**: Effects of protective behaviors were discussed. **Response costs**: Discussions about the response costs of protective behaviors against occupational hazards; for example, the difficulties of using masks for nurses. **Rewards of maladaptive responses**: Discussion about the rewards of maladaptive behaviors; For example, shaking hands with others will increase social interactions (This should not be done with contaminated hands).	First author		
3 (60 min)	Ergonomic and psychosocial hazards were discussed in more detail, and protective strategies against these hazards were educated.	Similar to the second session, but for ergonomic and psychosocial hazards.	First author		

The educational intervention in this study was implemented in three sessions (one session per week). At first, the educational content was presented face to face (lecture, Q&A, PowerPoints, PDF files), and then the PowerPoint slides and educational pamphlets were delivered to nurses in a way that was more convenient to them via their cellphones.

### Control group procedure

The control group did not receive any particular intervention during the study; the educational content was provided to those who were willing to receive it only after completing the study.

### Instruments

The instrument used to collect data consisted of two sections; a demographic characteristics form (13 items) and a scale for measuring emergency ward nurses’ protective behaviors against occupational hazards (39 items).

Demographic characteristics included age, sex, marital status, having children, education level (in nursing), work experience, types of work shifts, working in additional centers, working overtime, history of exposure to occupational hazards and diseases, suffering from underlying diseases, history of allergy to latex, and history of vaccination against potential occupational diseases.

The initial scale for measuring emergency ward nurses’ protective behaviors against occupational hazards was developed for this study by authors based on relevant literature [1–4, 14, 22] and the researchers’ experiences, and included 47 items. The initial scale’s face validity was assessed using qualitative and quantitative methods with ten nurses who had similar working conditions as the nurses participating in the study. The content validity was assessed using qualitative and quantitative methods such as the Content validity index (CVI) and Content validity ratio (CVR), by the participation of 15 occupational health experts and nursing professors and instructors. For the reliability of the scale, Cronbach’s alpha and Intraclass correlation coefficient (ICC) (a 2-week interval) were estimated by the participation of 20 nurses. Following this process out of the initial 47 items, 5 items were removed because CVR of items were less than 0.49 [23], and 3 items were removed due to covering the same concept according to the opinions of the experts and after the agreement of the research team. The item reduction process was carried out in a way such that the original content of the scale remained intact. The final scale included five sub-scales and 39 items, covering nurses’ protective behaviors against physical (items 1–6; scoring: 6–30), chemical (items 7–11; scoring: 5–25), biological (items 12–21; scoring: 10–50), ergonomics (items 22–26; scoring: 5–25), psychosocial (items 27–39; scoring: 13–65) and total hazards (items 1–39; scoring: 39–195). In order to better compare the subscales and the total score with each other, the mean score (1–5) of each was calculated. The items on the scale were scored based on a 5-point Likert scale (from Never (1) to Always (5)), and there was no reverse item. Higher scores indicated higher compliance with protective behaviors against occupational hazards (Supplementary File). All items obtained an impact score higher than 1.5, and the overall CVI was 0.96. After obtaining the necessary permits and providing some information about the objectives of the study, written informed consent was received from the participants. The nurses in both groups completed the demographic characteristics form and the scale designed to measure protective behaviors against occupational hazards before, immediately, and one month after the intervention. Cronbach’s alpha and ICC (a 2-week interval) of the scale among the 124 participants of this study were obtained as 0.930 and 0.832 respectively.

### Data analysis

Data were analyzed using descriptive (mean, Standard Deviation (SD), Mean Difference (MD), frequency, and frequency percentage) and inferential methods (Chi-square (χ2) or Fisher’s exact test, independent t-test, Analysis of Variance (ANOVA) and repeated measures ANOVA) in SPSS software (version 26; IBM Corp., Armonk, NY, USA). The assumptions of the repeated measure ANOVA included assumptions of normally, homogeneity of variance, homogeneity of covariances (sphericity), and no significant outliers were tested for nurses’ protective behaviors variables. These assumptions were established for underlying variables except sphericity assumption for some of the variables, which was modified by the Greenhouse-Geisser Correction. In the final analysis, to assess the intervention effect, we used the random effects model to allow for clustering design by considering a random effect for the clusters in the analysis. The significance level was set at *p* < 0.05.

## Results

The mean age of the participants was 33.79 ± 7.43 years, and the mean work experience was 8.55 ± 6.42 years. Most of the participants were female, married, and held Bachelor of Science (BSc) degrees in nursing. There were no statistically significant differences between the two groups in terms of demographic characteristics and groups were homogenous in terms of demographic variables, except for the types of work shifts (Table 2).

**Table 2:** Demographic characteristics of emergency ward nurses in the intervention (*n* = 62) and control (*n* = 62) groups

Demographic variables	Categories	Intervention *n*(%)	Control *n*(%)	Statistic test	*p*-value
Age	Years	33.47 ± 7.13^a^	34.11 ± 7.77^a^	t = − 0.481	0.631
Sex	Female	45(72.6)	52(83.9)	χ2 = 2.32	0.191
	Male	17(27.4)	10(16.1)		
Marital status	Single	30(48.4)	27(43.5)	*F* = 0.432	0.891
	Married	30(48.4)	33(53.2)		
	Divorced	2(3.2)	2(3.2)		
Having children	Yes	18(29)	26(41.9)	χ2 = 2.255	0.189
	No	44(71)	36(58.1)		
Education level (in nursing)	BSc	60(96.8)	54(87.1)	χ2 = 3.916	0.095
	MSc	2(3.2)	8(12.9)		
Work experience	Years	7.96 ± 5.97^a^	9.15 ± 6.84^a^	t = -1.028	0.306
Types of work shift	Morning	5(8.1)	12(19.4)	*F* = 9.520	0.014 ^*^
	Night	1(1.6)	1(1.6)		
	Evening and night.	2(3.2)	9(14.5)		
	In circulation	54(87.1)	40(64.5)		
Working in additional centers	Yes	5(8.1)	5(8.1)	χ2 = 0.000	1
	No	57(91.9)	57(91.9)		
Working overtime	Yes	61(98.4)	60(96.8)	*F*=-	1
	No	1(1.6)	2(3.2)		
History of exposure to occupational hazards and diseases	Yes	31(50)	28(45.2)	χ2 = 0.291	0.719
	No	31(50)	34(54.8)		
Suffering from underlying diseases	Yes	11(17.7)	6(9.7)	χ2 = 1.704	0.296
	No	51(82.3)	56(90.3)		
History of allergy to latex	Yes	11(17.7)	18(29)	χ2 = 2.205	0.203
	No	51(82.3)	44(71)		
History of vaccination against potential occupational diseases	HBV	4(6.5)	5(8.1)	*F* = 1.655	0.657
	COVID-19	18(29)	19(30.6)		
	HBV & COVID-19	21(33.9)	25(40.3)		
	HBV & COVID-19 & influenza	19(30.6)	13(21)		

Note: ^*^Significant at *p* < 0.05

^a^ Mean ± SD

BSc = Bachelor of Science; MSc = Master of science; HBV = Hepatitis B virus; t = independent t-test; χ2 = Chi square test; F = Fisher’s exact test

The results of the independent samples t-test showed that the mean scores of protective behaviors against ergonomic and psychosocial hazards were not statistically significant (*p* > 0.05) between the control and intervention groups before the intervention; however, the mean scores of protective behaviors against physical, chemical, biological and total hazards were significantly higher in the control group than in the intervention group at the baseline (*p* < 0.05). Immediately and one month after the educational intervention, the mean scores of protective behaviors in all dimensions were significantly higher in the intervention group than in the control group (*p* < 0.05); except for the physical hazard sub-scale measured immediately after the intervention (t = 1.342, *p* = 0.182) (Table 3).

Intragroup comparison using the one-way repeated measure ANOVA showed a significant increase in the total mean score of protective behaviors and sub-scales in the intervention group over time, reflecting the impact of the educational intervention on the protective behaviors of nurses in the intervention group, while a declining trend was noticed in the control group over time (Table 3). Bonferroni post-hoc comparison procedure indicated that at the measurements of pre-intervention, immediately and one-month after the intervention, the total mean scores of protective behaviors against occupational hazards and all sub-scales were statistically significant differences in the intervention and control groups (*p* < 0.05); except for physical (MD = 0.075, *p* = 0.089) and ergonomic hazards (MD = 0.023, *p* = 1) measured at pre-intervention and immediately after the intervention in the control group, as well as for psychosocial hazards (MD = 0.046, *p* = 0.056) in the control group and ergonomic hazards (MD=-0.071, *p* = 0.461) in the intervention group measured immediately and one month after the intervention.

**Table 3:** Comparison of the total and sub-scales mean score of nurses’ protective behaviors against occupational hazards in the intervention and control groups over time

Nurses’ protective behaviors against …	Time point	Group		t(*p*-value)
		Intervention	Control	
		Mean (SD)	Mean (SD)	
Total hazards	Pre-intervention	3.39(0.52)	3.58(0.51)	-2.082 (0.039) ^*^
	Immediately after intervention	3.83(0.38)	3.48(0.44)	4.714 (< 0.001) ^*^
	One-month after intervention	3.99(0.27)	3.41(0.40)	9.427 (< 0.001) ^*^
F (df time, df error) (*p*-value)		113.417 (1.64, 100.48) (< 0.001) ^*^	47.293 (1.39, 85.06) (< 0.001) ^*^	
Interaction of time and group by mixed ANOVA				
F (df time×group, df error) (*p*-value)		155.28 (1.61, 196.52) (< 0.001)^*^		
Physical hazards	Pre-intervention	3.57(0.58)	3.83(0.63)	-2.354 (0.020) ^*^
	Immediately after intervention	3.89(0.53)	3.75(0.60)	1.342 (0.182)
	One-month after intervention	4.06(0.32)	3.67(0.52)	5.009 (< 0.001) ^*^
F (df time, df error) (*p*-value)		45.03 (1.91, 116.9) (< 0.001) ^*^	10.55 (1.61, 98.62) (< 0.001) ^*^	
Interaction of time and group by mixed ANOVA				
F (df time×group, df error) (*p*-value)		54.44(1.86, 227.35) (< 0.001)^*^		
Chemical hazards	Pre-intervention	3.13(0.81)	3.51(0.68)	-2.585 (0.005) ^*^
	Immediately after intervention	3.67(0.56)	3.42(0.66)	2.242 (0.027) ^*^
	One-month after intervention	3.99(0.42)	3.36(0.65)	6.316 (< 0.001) ^*^
F (df time, df error) (*p*-value)		68.652 (1.77, 108.08) (< 0.001) ^*^	17.377 (1.81, 110.85) (< 0.001) ^*^	
Interaction of time and group by mixed ANOVA				
F (df time×group, df error) (*p*-value)		84.53 (1.78, 216.98) (< 0.001)^*^		
Biological hazards	Pre-intervention	3.80(0.56)	4.02(0.52)	-2.250 (0.026) ^*^
	Immediately after intervention	4.14(0.37)	3.82(0.45)	4.258 (< 0.001) ^*^
	One-month after intervention	4.30(0.25)	3.71(0.45)	8.725 (< 0.001) ^*^
F (df time, df error) (*p*-value)		66.954 (1.37, 83.89) (< 0.001) ^*^	52.407 (1.46, 89.46) (< 0.001) ^*^	
Interaction of time and group by mixed ANOVA				
F (df time×group, df error) (*p*-value)		117.99 (1.4, 171.66) (< 0.001)^*^		
Ergonomic hazards	Pre-intervention	2.86(0.76)	2.90(0.70)	-0.293 (0.770)
	Immediately after intervention	3.55(0.41)	2.88(0.62)	7.064 (< 0.001) ^*^
	One-month after intervention	3.62(0.39)	2.80(0.53)	9.805 (< 0.001) ^*^
F (df time, df error) (*p*-value)		64.930 (1.47, 90.23) (< 0.001) ^*^	5.277 (1.63, 99.54) (0.006) ^*^	
Interaction of time and group by mixed ANOVA				
F (df time×group, df error) (*p*-value)		64.93 (1.52, 185.67) (< 0.001)^*^		
Psychosocial hazards	Pre-intervention	3.28(0.64)	3.41(0.69)	-1.084 (0.280)
	Immediately after intervention	3.74(0.46)	3.35(0.59)	3.99 (< 0.001) ^*^
	One-month after intervention	3.87(0.36)	3.31(0.54)	6.78 (< 0.001) ^*^
F (df time, df error) (*p*-value)		67.549 (1.52, 92.81) (< 0.001) ^*^	9.827 (1.60, 98.14) (< 0.001) ^*^	
Interaction of time and group by mixed ANOVA				
F (df time×group, df error) (*p*-value)		76.58 (1.53, 187.81) (< 0.001)^*^		

Note: ^*^Significant at *p* < 0.05

## Discussion

The present study aimed to evaluate the effects of an educational intervention based on the protection motivation theory on the protective behaviors of emergency ward nurses against occupational hazards. The findings showed that nurses in the intervention and control groups were similar in terms of demographic characteristics. Most nurses were female, married, without children, and had BSc degrees. Most of the participants just worked in one hospital, and had a history of vaccination against HBV and COVID-19. Most of the nurses had no history of allergy to latex, had no underlying disease, and had no history of exposure to occupational hazards and diseases. The results of this study indicated that the PMT-based educational intervention improved the emergency ward nurses’ protective behaviors against various types of occupational hazards (physical, chemical, biological, ergonomics, and psychosocial hazards) in the intervention group. The results of a study in Iran showed that training a standard guideline about the safe handling of antineoplastic drugs, effectively improved the knowledge and behaviors of chemotherapy ward nurses [24]. Another study in Iran, showed that efficacy, effectiveness and rewards were the most predictors constructs of PMT for adherence to safe injection guidelines among nurses, suggesting that educational interventions for nurses should be more focused on these constructs [25]. In the present study, we included the most important constructs of the PMT for preparing and delivering the education content to emergency ward nurses. Another study in India also revealed that educational workshops improved HCWs’ knowledge about occupational hazards [26]. A literature review study also highlighted the positive impacts of e-training programs on employees’ knowledge and behavior regarding occupational health and safety and reducing workplace injuries [27]. These findings are consistent with the present study regarding the impact of educational intervention on individuals’ protective behaviors against occupational hazards, also it should be denoted that some parts of the educational intervention in the present study were delivered virtually on mobile platforms. A study on the efficiency of web-based learning in preventing exposure to occupational hazards in a clinical nursing setting showed that this type of education could significantly boost knowledge, but no remarkable changes were seen with regard to attitudes and behaviors [28]. Regarding behavioral dimensions, our results differed from the above-mentioned survey, which could be due to differences in training methods and educational content used in these studies. The present study used multi-methods approaches for education such as face-to-face and virtual methods, whereas education in the above-mentioned study was purely web-based. It should also be noted that changes in behavior are not solely dependent on knowledge, and other factors such as workload, time availability, access to facilities, and self-efficacy may also be influential. For instance, a study identified that type of profession, self-efficacy and behavioral intention were related factors to HCWs’ protective behaviors against COVID-19 [22]. In the present study, education was based on the constructs of PMT, and various factors for changing protective behaviors were discussed with the participants. Anyway, education is considered an effective factor for changing the behaviors of people in other topics and contexts [15–18].

A study investigated the impact of an educational program on overall occupational safety and ergonomic, biological, radiation, and chemical hazards among nurses and other HCWs in India and verified the influence of this program in boosting knowledge regarding these hazards. The effect of this program on the knowledge of biological hazards was highest and for the radiation, and chemical hazards were lowest. The participants of the recent study suggested that psychosocial hazards should be added to educational programs [29]. In the present study, psychosocial hazards were also considered. We also observed that in the intervention group and one month after the intervention, the highest and lowest mean scores of protective behaviors belonged to biological and ergonomic hazards, respectively. The results of the present study were consistent with the findings of the above-mentioned study on biological hazards and inconsistent with regard to ergonomic hazards. This discrepancy may be related to factors such as the educational content, nurses’ self-efficacy and access to equipment for performing protective behaviors against occupational hazards.

### Limitation

There are several limitations to consider in this study. The construct validity of the scale of emergency nurses’ protective behaviors against occupational hazards was not investigated and verified. This scale was a self-report, so the data in some dimensions might not reflect the actual levels of nurses’ protective behaviors. Future studies can use more objective scales for evaluating these behaviors. Additionally, the participants in this study were selected from educational and public hospitals, which might limit the generalizability of results to nurses working in private hospitals. There could be some organizational factors such as rules and laws that were not evaluated in this study, so future studies can also pay attention to these factors. Finally, data collection was conducted immediately and one month after the intervention, so longer follow-ups (3–6 months) are recommended for future studies to determine the durability of protective behaviors and the long-term effects of the educational intervention.

## Conclusion

The study results showed that the implementation of an educational intervention based on PMT constructs could be effective and valuable in increasing the protective behaviors of emergency nurses against occupational hazards. Education alone is insufficient to change nurses’ health behaviors against occupational hazards. More attention should be paid to other factors affecting health and protective behaviors, such as access to personal protective equipment (PPE), work conditions, facilities, organizational regulations, state rules and laws, workload, and time restrictions. Future research efforts can be focused on designing more specific educational interventions based on the needs of nurses and also include nurses from various hospital wards.

### Electronic supplementary material

Below is the link to the electronic supplementary material.

Supplementary Material 1: The online version contains a supplementary file (The Scale of emergency ward nurses’ protective behaviors against occupational hazards)

## Data Availability

The datasets used and/or analyzed during the current study are available from the corresponding author on reasonable request.

## Abbreviations

ANOVA: Analysis of variance
BSc: Bachelor of Science
CVI: Content Validity Index
CVR: Content Validity Ratio
HBV: Hepatitis B virus
HCV: Hepatitis C virus
HCWs: HealthCare Workers
HIV: Human immunodeficiency virus
ICC: Intraclass Correlation Coefficient
MD: Mean Difference
MSc: Master of science
PMT: Protection Motivation Theory
SD: Standard Deviation
SPSS: Statistical Package for the Social Sciences
USA: United States of America
χ2: Chi-square
