# Supplementary material for: The effects of an educational intervention based on the protection motivation theory on the protective behaviors of emergency ward nurses against occupational hazards: a quasi-experimental study
Source: BMC Nurs. 2024 Jun 18;23:409. doi: 10.1186/s12912-024-02053-1 (PMC11184892; doi:10.1186/s12912-024-02053-1)
Supplement: Supplementary file 1 — Supplementary Material 1: The online version contains a supplementary file (The Scale of emergency ward nurses’ protective behaviors against occupational hazards) [file 12912_2024_2053_MOESM1_ESM.docx]

| **Supplementary File.** The scale of emergency ward nurses' protective behaviors against occupational hazards | | | |
| --- | --- | --- | --- |
| **Subscales of protective behaviors of nurses against...** | **Items**  **How often do you do the following behaviors in your workplace?** | **Cronbach's alpha** | **ICC** |
| **Physical hazards (6 items) (1-6)** | 1. I wear suitable shoes (standard and slip-resistant shoes). | 704.0 | 863.0 |
|  | 2. I am careful when using sharp objects (such as blades and needles). |  |  |
|  | 3. I do not use faulty medical and electrical devices with potential risks of physical hazards (e.g., electrocution, etc.). |  |  |
|  | 4. I avoid exposure to radiation (such as X-ray, UV, etc.). |  |  |
|  | 5. I avoid exposure to extreme temperatures. |  |  |
|  | 6. I avoid exposure to extreme sound and noise. |  |  |
| **Chemical hazards (5 items) (7-11)** | 7. I use suitable hand creams and lotions to prevent contact dermatitis. | 749.0 | 839.0 |
|  | 8. I wear a face mask when in contact with cleaning, disinfecting, and sterilizing materials. |  |  |
|  | 9. I avoid being in places that have been cleaned recently with cleaning or disinfectant materials. |  |  |
|  | 10. I avoid direct contact of hands with cleaning, disinfecting, and sterilizing materials. |  |  |
|  | 11. I avoid contact with materials that I am allergic to (e.g., latex gloves and other equipment containing latex, etc.). |  |  |
| **Biological hazards (10 items) (12-21)** | 12. I do hand hygiene and handwashing properly. | 793.0 | 837.0 |
|  | 13. I don’t recap needles after procedures. |  |  |
|  | 14. I correctly discard infectious and non-infectious waste and sharp objects. |  |  |
|  | 15. I follow the principles of isolation when necessary. |  |  |
|  | 16. I disinfect or make sure that surfaces that I have the most contact with are disinfected (e.g., desk, mobile phone, keys, etc.) |  |  |
|  | 17. I maintain a minimum distance of 1 m from others during the outbreak of respiratory infectious diseases (such as Influenza or COVID-19). |  |  |
|  | 18. I wear a face mask when in contact with individuals who have respiratory infectious diseases, signs, and symptoms. |  |  |
|  | 19. I Use PPE (e.g., gloves, glasses, eye protection, disposable gowns, shoe covers, head covers) as required. |  |  |
|  | 20. I provide proper ventilation (natural or artificial ventilation without air recirculation) as required. |  |  |
|  | 21. I avoid going home with work clothes. |  |  |
| **Ergonomic hazards (5 items) (22-26)** | 22. I seek assistance from my colleagues or use special equipment for moving and changing the position of the patients. | 776.0 | 776.0 |
|  | 23. I do stretching exercises before, during, and after the work shift. |  |  |
|  | 24. I have the right body posture while performing tasks and duties. |  |  |
|  | 25. I properly sit on a chair or while working with a computer. |  |  |
|  | 26. I take a brief rest after feeling tired from doing a task in a particular position (e.g., standing). |  |  |
| **Psychosocial hazards (13 items) (27-39)** | 27. I provide conditions for having enough and higher quality sleep (e.g., a suitable and quiet sleeping environment, avoiding using mobile phones, etc.). | 877.0 | 873.0 |
|  | 28. I refer to a psychologist, supervisor, or other experts to reduce job stress, and burnout if needed. |  |  |
|  | 29. I separate work issues from personal life. |  |  |
|  | 30. I care for my own physical and mental health. |  |  |
|  | 31. I have a suitable and complete diet. |  |  |
|  | 32. I have enough rest on some days (going on vacation and resting). |  |  |
|  | 33. I avoid overtime and working at multiple centers as much as possible. |  |  |
|  | 34. I use ways to reduce exposure to violence at the workplace from patients and their companions. |  |  |
|  | 35. I pursue workplace violence with the hospital officials if necessary. |  |  |
|  | 36. I use strategies to control anger (e.g., deep breathing, speaking calmly, etc.). |  |  |
|  | 37. I use prescribed restraints or medications for aggressive patients. |  |  |
|  | 38. I seek help from others if I am at risk of workplace violence or potential harm. |  |  |
|  | 39. If I am going to do something alone in an environment, my colleagues will be able to see my location or know my location (for prevention of workplace violence). |  |  |
